# Auditory perception modulated by word reading

**DOI:** 10.1007/s00221-016-4706-5

**Published:** 2016-06-21

**Authors:** Liyu Cao, Anne Klepp, Alfons Schnitzler, Joachim Gross, Katja Biermann-Ruben

**Affiliations:** 1 School of Psychology, University of Glasgow, Glasgow, G12 8QB UK; 2 Institute of Clinical Neuroscience and Medical Psychology, Medical Faculty, Heinrich Heine University Düsseldorf, Düsseldorf, 40225 Germany; 3 Institute of Neuroscience and Psychology, University of Glasgow, Glasgow, G12 8QB UK

**Keywords:** Embodied cognition, Auditory perception, Language processing, Sound-related words

## Abstract

Theories of embodied cognition positing that sensorimotor areas are indispensable during language comprehension are supported by neuroimaging and behavioural studies. Among others, the auditory system has been suggested to be important for understanding sound-related words (visually presented) and the motor system for action-related words. In this behavioural study, using a sound detection task embedded in a lexical decision task, we show that in participants with high lexical decision performance sound verbs improve auditory perception. The amount of modulation was correlated with lexical decision performance. Our study provides convergent behavioural evidence of auditory cortex involvement in word processing, supporting the view of embodied language comprehension concerning the auditory domain.

## Introduction

According to theories of embodied cognition, language comprehension involves a distributed brain network, including sensorimotor areas—at least for concrete words (Barsalou 2008; Pulvermüller 1999, 2013). Accumulating evidence from neuroimaging studies supports this idea. For example, in an fMRI study Hauk et al. (2004) showed a somatotopically organized activation in the motor and premotor cortex when participants passively read action-related verbs, i.e. leg-related action words lead to activations more medially than arm- or face-related action words. A recent MEG study replicated this finding and found that the activation in the motor cortex could be detected about 80 ms after the onset of the full word, thus ruling out the possibility that the observed activation in the motor cortex is a result of mental imagery and supporting the idea of embodied language comprehension (Shtyrov et al. 2014). In addition to neuroimaging studies, behavioural studies showed that motor responses (reaction time (RT), accuracy) were influenced after reading action-related words (Andres et al. 2015; Boulenger et al. 2008; Klepp et al. 2015; Mirabella et al. 2012; Sato et al. 2008). For example, Mirabella et al. (2012) found increased RTs and increased error rates in motor responses in a Go/NoGo task when the same effector was involved as in the presented verbs.

Besides the findings from the motor area, domain-specific activation during language comprehension in olfactory, gustatory and auditory brain areas was also found (Barrós-Loscertales et al. 2002; González et al. 2006; Kiefer et al. 2008). Kiefer et al. (2008) found stronger activation in the auditory brain area when participants read words that were sound related compared to words that were not sound related. In a single case report, a patient with focal lesions in the left auditory area demonstrated deficits in processing words depicting sound-related everyday objects, e.g. a bell (Trumpp et al. 2013a). However, unlike in the motor domain, behavioural effects in sensory domains are rarely reported. The aim of this study is to investigate whether auditory perception is modulated when participants read verbs related to sound, to sound plus action, or to neither sound nor action.

To assess auditory perception, a sound detection task was used, in which participants were required to indicate whether a near-threshold tone was presented or not. We hypothesize a priming effect on auditory perception after reading sound-related words (i.e. an enhanced detection performance), drawing on the finding that the auditory area is involved in the comprehension of visually presented sound-related words (Kiefer et al. 2008). For words that are related to both sound and action, we predict a cross-domain effect on auditory perception from the motor involvement, which adds on the auditory priming effect hypothesized above. The rationale of this hypothesis is that a suppressive pathway from the motor cortex to the auditory cortex was reported in human and animal studies. Martikainen et al. (2005) showed that brain responses to a simple tone were smaller when the tone was triggered by a button press of the respective participant than when the tone was externally controlled by the computer. Recent animal studies suggested a motor origin of this suppression effect (Schneider et al. 2014). We predict that covert activation of motor/premotor brain areas generated by reading (sound-plus-) action-related words should have a comparable suppression effect on auditory perception. This would result in a reduced detection performance as compared to purely sound-related verbs.

Additionally, behavioural studies of action-related words on motor responses suggested that the stimulus onset asynchrony (SOA) between prime verb and Go signal has an effect on behavioural results, with a short SOA (100 ms) leading to an interference effect and a long SOA (350 ms) leading to a facilitation effect (de Vega et al. 2013). We included a shorter (50 ms) and a longer (300 ms) latency between the offset of the stimulus word and the onset of the sound detection task to explore this factor in the auditory domain.

## Methods

### Participants

Thirty participants (mean age: 26.4; age range 18–44; 17 females; 1 left-handed) were recruited from university campus. All participants were native German speakers and reported normal hearing. Informed consent was obtained prior to the experiment. Participants were debriefed and received monetary compensation after the experiment. The experiment was conducted in accordance with the Declaration of Helsinki and was approved by Düsseldorf University ethics committee (study number 3400).

### Word stimuli

First we collected 54 purely sound-related verbs, 96 sound plus mouth action-related verbs and 48 abstract verbs with neither sound nor action content. Choosing mouth action verbs instead of hand action verbs allowed us to compare manual RT between different verb conditions without interference between verb content and response effector. These verbs will further be labelled *sound plus action* verbs. This initial selection of verbs for the three categories was assessed in a multistep rating, exclusion and matching procedure to define suitable experimental stimuli. To this end, 35 participants were asked to rate sound relatedness, action relatedness and familiarity of all verbs in a randomized online questionnaire (35 participants completed the sound relatedness judgment; 28 the action relatedness judgment; 28 the familiarity judgment. None of them participated in the main behavioural study). Participants rated from 1 (very weak) to 6 (very strong), indicating to what extent they associate the word with a sound (sound relatedness), to what extent the action depicted by the word is executed with physical strength and/or amplitude (action relatedness) and to what extent they are familiar with the word (familiarity). For action relatedness, the scale included the option ‘not an action’, indicating that the verb is not associated with any kind of action that a person can execute. ‘Not an action’ was coded as 0 in addition to the options 1–6 for sound verbs and sound plus action verbs. All abstract verbs were taken from former studies (Klepp et al. 2014; Niccolai et al. 2014) where the ratings did not include this option since all verbs were executable actions—either abstract or concrete. The data were collected online with SoSci Survey (https://www.soscisurvey.de/). Finally, we selected 16 sound verbs that were related to sound (mean rating = 4.73; SD = 0.70) but not to action (mean rating = 1.10; SD = 0.41; ‘not an action’ ratio: 0.64), 16 sound plus action verbs that were related to both sound (mean rating = 4.83; SD = 0.50) and action (mean rating = 3.34; SD = 0.59; ‘not an action’ ratio: 0) and 16 abstract verbs that were not related to sound (mean rating = 1.19; SD = 0.10) or action (mean rating = 1.26; SD = 0.17; see “Appendix” for a list of all the words used). We failed to find enough words that are (mouth) action related but not sound related. Independent sample t tests showed that sound plus action verbs had higher action relatedness than both sound verbs (*t*(30) = 12.08, *p* < 0.001) and abstract verbs (*t*(30) = 13.18, *p* < 0.001, *p* < 0.001). Moreover, sound plus action verbs (*t*(30) = 27.39, *p* < 0.001) and sound verbs (*t*(30) = 19.44, *p* < 0.001) had higher sound relatedness than abstract verbs. No other t tests reached significance. For each word, we also obtained word frequency (Biemann et al. 2007), bigram frequency and trigram frequency (Baayen et al. 1995), the latter two of which were calculated as the mean frequency of all relevant units in the word (e.g. bigrams in the word ‘surren’ include ‘su’, ‘ur’, ‘rr’, ‘re’ and ‘en’) from the database. Word frequency was different among verbs (*F*(2,45) = 5.00; *p* = 0.01). Independent sample *t* tests showed that sound plus action verbs (mean = 16.50; SD = 1.41) have a lower frequency than abstract verbs (mean = 14.63; SD = 1.54) (*t*(30) = 3.48, *p* = 0.002) (note that a higher frequency class value indicates a lower word frequency). No differences were present in frequency between sound verbs (mean = 15.69; SD = 1.89) and sound plus action verbs (*t*(30) = −1.33, *p* = 0.19) or between sound verbs and abstract verbs (*t*(30) = 1.69, *p* = 0.10). Despite the differences in frequency, all three selected categories of words were matched for familiarity (*F*(2,45) = 1.88; *p* = 0.16), word length (*F*(2,45) = 1.57; *p* = 0.22), bigram frequency (*F*(2,45) = 0.57; *p* = 0.57) and trigram frequency (*F*(2,45) = 1.63; *p* = 0.21). Table 1 provides a summary of the above mentioned word parameters.

**Table 1:** Means and standard deviation (in brackets) of related word parameters

	Sound plus action verbs	Sound verbs	Abstract verbs
Sound relatedness	4.83 (0.50)	4.73 (0.70)	1.19 (0.10)
Action relatedness	3.34 (0.59)	1.10 (0.41)	1.26 (0.17)
Familiarity	5.68 (0.20)	5.51 (0.30)	5.66 (0.30)
Word length	7.69 (1.25)	7.19 (1.38)	6.94 (1.00)
Word frequency	16.50 (1.41)	15.69 (1.89)	14.63 (1.54)
Bigram frequency	12,940.91 (1913.91)	12,398.25 (2255.83)	12,025.23 (3034.00)
Trigram frequency	4531.27 (1859.08)	4022.69 (2443.22)	3098.41 (2478.61)

Note that for word frequency, higher values indicate lower frequencies

For the lexical decision task, 48 pseudowords were generated as counterparts to the selected word stimuli using Wuggy, a multilingual pseudoword generator (Keuleers and Brysbaert 2010). Given a German word as input, it automatically generates a list of counterpart pseudowords that are matched for subsyllabic structure and transition frequencies.

### Procedure

In a pretesting phase, the 75 % detection threshold for a 1000 Hz tone (100 ms duration; 5 ms rise/fall) was individually identified for each participant, specifying the stimulus intensity to be used in the main task. To control for environmental noise, a background noise set at a comfortable level was presented throughout the whole experiment (including the main task). The test tone was presented in five different intensities (−29, −26, −23, −20, −17 dB in reference to background noise), and participants were required to judge whether they heard a tone or not. Each intensity was presented 36 times in random order.

**Fig. 1:**
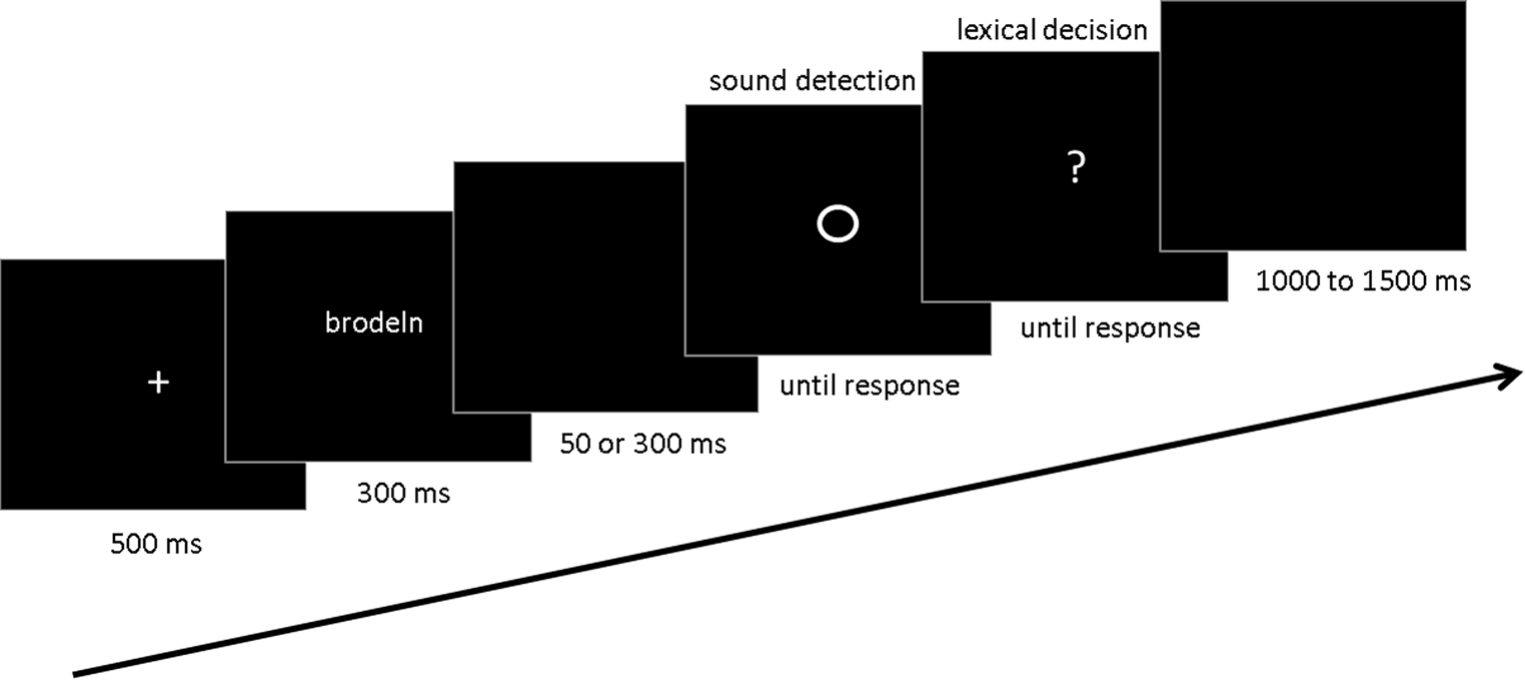
Main task. Each trial starts with a fixation cross for 500 ms, which is followed by a word stimulus for 300 ms (in this example, ‘brodeln’, ‘to seethe’ in English). After a short (50 ms) or long (300 ms) latency, a *circle* is presented. In half of the trials, the circle is presented together with a test tone which the participants are required to detect as quickly and as accurately as possible (sound detection task). A question mark follows after the sound detection response prompting the participant to respond whether the *word* shown in the beginning of the trial is a real word or not (lexical decision task). This is followed by a random inter-trial interval between 1000 and 1500 ms

In the main task (Fig. 1), participants sat in front of a computer screen at a viewing distance of approximately 50 cm. A trial began with the presentation of a fixation cross in the centre of the screen for 500 ms. After that, a word stimulus was centrally presented for 300 ms. Participants were required to silently read the word in order to judge whether it was a real word or a pseudoword at the end of the trial. After a short (50 ms) or long latency (300 ms) during which the screen remained blank, a circle appeared in the centre of the screen. In half of the trials, the circle was accompanied by the test tone with the intensity determined in the pretest. Participants were required to respond as accurately and as quickly as possible whether they had heard a tone. After the response, they were given the lexical decision task, judging whether the word presented at the beginning of the trial was a real word or a pseudoword. This was aimed to make participants pay attention to the word stimuli. After the participant’s response, the next trial started with a jittered interval between 1000 and 1500 ms.

Each real word stimulus was presented 8 times in total, equally divided between short and long latency, sound stimulus present and sound stimulus absent. The pseudowords were presented once each, resulting in 432 trials in total. All (pseudo-) word stimuli were presented in random order. The experiment was run in 4 blocks, each containing 108 trials. Participants used a keyboard to respond. The assignments of responses and buttons were counterbalanced between participants in the sound detection task: half of the participants pressed the left arrow button for tone present, the right arrow button for tone absent and vice versa. The lexical decision was always reported with button ‘A’ for word and button ‘D’ for pseudoword. All sound stimuli were delivered through a pair of headphones (Sony MDR-XD100), and the experiment was run with Psychtoolbox-3 on Matlab (The MathWorks Inc., Natick, MA, USA). The duration of the study was about 70 min for each participant including pretest and main experiment.

### Data analysis

For the pretesting phase data, we obtained a detection probability for each of the five tone intensities. Then, the five data points were plotted on a Cartesian coordinate system with tone intensity on the x-axis and detection probability on the y-axis. At last, a sigmoid curve was fitted to the five data points and 75 % detection threshold was determined (the same method as used in Borra et al. 2013).

For the main task data, one participant had to be excluded from further analysis due to very low accuracy (0.58) in the lexical decision task. For all other participants, trials starting with pseudowords were not analysed. Trials with incorrect answers in the lexical decision task were also excluded. In these trials, either no real verbs were presented or participants were failed to recognize the verbs, thus excluding a conclusive sensory modulation causally related to verb processing. Trials that were followed by RTs exceeding 3 standard deviations of the individual mean RT in the sound detection task were excluded. 83.1 % (SD = 3.8 %) trials remained for the final analysis. The accuracy and RT data in the sound detection task were submitted to a 2 (latency: long and short) by 3 (word category: sound, sound plus action and abstract verbs) repeated-measures ANOVA. In addition, planned comparisons were carried out by means of paired t tests to follow up significant main effects or interactions. We also grouped the participants into two subgroups based on their performance in the lexical decision task and then performed a 2 (group: HP and LP) by 2 (latency: long and short) by 3 (word category: sound, sound plus action and abstract verbs) mixed-design ANOVA analysis with the data. The rationale is that participants with higher accuracy in the lexical decision task were more attentive to the word stimulus and thus might have processed the word at deeper levels. Level of processing has been shown to be important in a language–motor interaction study (Sato et al. 2008). The modulation effect in the sound detection task, if present, should be more likely to be observed in participants with better lexical decision performance. The 29 participants were divided into a high-performance (HP) group and a low-performance (LP) group. The HP group consists of the first 15 participants in the lexical decision accuracy ranking. Their mean accuracy resulted in 0.98 (SD = 0.01). The LP group consists of the other 14 participants according to the accuracy ranking. Their mean accuracy resulted in 0.91 (SD = 0.05). Additionally, a sound detection performance modulation effect was calculated. Since a higher tone detection performance was predicted for *sound* verbs than both *sound plus action* verbs and *abstract* verbs, the detection accuracy following both verbs was subtracted from the detection accuracy following sound verbs separately, for both short and long latency conditions. The average of the resulting four (2 verb categories × 2 latencies) values was then taken as the modulation effect, indexing the strength of the modulation of auditory perception after reading sound words. A positive modulation effect emerges in case a participant on average benefits from sound verbs as opposed to the other verb categories with regard to tone detection accuracy. A negative modulation effect indicates that on average a participant’s performance declines after sound verbs presentation. We performed a correlation analysis between the modulation effect and accuracy in the lexical decision task across participants who showed a positive modulation effect. The ANOVA analysis was conducted with SPSS 19, and the correlation analysis was performed with the robust correlation toolbox (Pernet et al. 2012) implemented in Matlab.

## Results

### Accuracy of tone detection

**Fig. 2:**
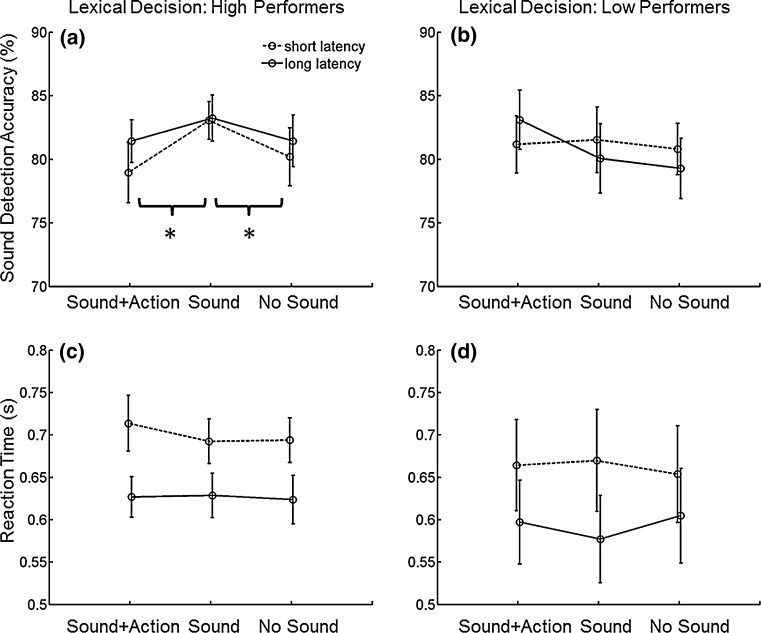
Accuracy (*upper row*) and reaction times (*lower row*) in the sound detection task (mean ± standard error of mean) separated for participants with high (>96 % correct, mean = 98 %; left column) and low (<96 % correct, mean = 91 %; right column) lexical decision performance. For high lexical decision performers, accuracy for sound verbs is significantly higher than for sound/action and abstract (no sound) verbs. The pattern of results is comparable but accentuated for short as compared to long latency condition. This effect is not seen in the low lexical decision performers (*upper right panel*). Reaction time is not modulated by word category in either participant group

The repeated-measures ANOVA analysis with all 29 participants revealed no significant effects (word category: *F*(2,56) = 1.64, *p* = 0.21; latency: *F*(1,28) = 0.36, *p* = 0.55; interaction: *F*(2,56) = 2.07, *p* = 0.14). When participants were grouped based on the lexical decision performance, a 2 (group) by 2 (latency) by 3 (word category) mixed-design ANOVA analysis revealed a significant interaction effect between group and word category (*F*(2,54) = 3.41, *p* = 0.04; sphericity assumed). Post hoc analysis showed that there was a significant modulation of the tone detection accuracy across word categories in the lexical decision high-Performance group (*F*(2,28) = 4.53, *p* = 0.02; Fig. 2a), with the accuracy after reading sound verbs being higher than the accuracy after reading sound plus action verbs (*t*(14) = 2.42, *p* = 0.03) and abstract verbs (*t*(14) = 2.33, *p* = 0.04). No such modulation was found in the low-performance group (*F*(2,26) = 1.21, *p* = 0.31; Fig. 2b). No other effects from the mixed-design ANOVA analysis reached statistical significance [group: *F*(1,27) = 0.02, *p* = 0.88; latency: *F*(1,27) = 0.32, *p* = 0.58; word category: *F*(2,54) = 1.71, *p* = 0.19; group vs. latency: *F*(1,27) = 0.98, *p* = 0.33; latency vs. word category: *F*(2,54) = 2.05, *p* = 0.14; group vs. latency vs. word category: *F*(2,54) = 0.28, *p* = 0.76].

A control analysis was performed to compare lexical decision performance among word categories with a 2 (group) by 3 (word category) mixed-design ANOVA. This revealed significant main effects of group (*F*(1,27) = 29.78, *p* < 0.001) and word category (*F*(2,54) = 8.38, *p* = 0.002; Greenhouse–Geisser correction) and also a significant interaction effect (*F*(2,54) = 4.24, *p* = 0.03; Greenhouse–Geisser correction). The main effect of group with lexical decision performance is not surprising given the group categorization procedure. The main effect of word category was followed by a post hoc analysis, which showed that the lexical decision performance was significantly lower following sound verbs than following sound plus action verbs (*t*(28) = −2.83, *p* = 0.01) and abstract verbs (*t*(28) = −2.95, *p* = 0.01). However, the significant interaction effect showed that the modulation of lexical decision performance across word categories was only true for the LP group (*F*(2,26) = 6.33, *p* = 0.01), but not for the HP group (*F*(2,28) = 2.09, *p* = 0.16) (see Table 2 for the lexical decision accuracy data for each group). Post hoc paired t tests within the LP group showed that sound verbs led to significantly lower lexical decision accuracy than sound plus actions verbs (*t*(13) = −3.00, *p* = 0.01) and abstract verbs (*t*(13) = −2.71, *p* = 0.02).

**Table 2:** Lexical decision accuracy across word categories for both HP and LP groups (with standard deviation in brackets)

	Sound plus action verbs	Sound verbs	Abstract verbs
HP group	98.23 % (1.3 %)	97.66 % (2.2 %)	98.80 % (0.9 %)
LP group	93.30 % (4.7 %)	88.45 % (7.9 %)	94.42 % (2.8 %)

A mixed-design ANOVA analysis shows a main effect that the HP group has higher accuracy than the LP group. An interaction effect indicates that, in the LP group sound verbs lead to significantly lower accuracy than the other two word categories, the pattern of which is not present in the HP group

### RT of tone detection

RT data were analysed similarly with a 2 (group) by 2 (latency) by 3 (word category) mixed-design ANOVA as for the accuracy data. There was a significant main effect of latency (*F*(1,27) = 58.01, *p* < 0.001), with participants responding faster in the long latency condition (mean = 610 ms, SD = 151) than in the short latency condition (mean = 682 ms, SD = 161). No other effects reached statistical significance [group: *F*(1,27) = 0.38, *p* = 0.55; word category: *F*(2,54) = 0.69, *p* = 0.45 (Greenhouse–Geisser correction); group vs. latency: *F*(1,27) = 0.05, *p* = 0.83; group vs. word category: *F*(2,54) = 0.24, *p* = 0.79; latency vs. word category: *F*(2,54) = 0.85, *p* = 0.43; group vs. latency vs. word category: *F*(2,54) = 1.58, *p* = 0.22].

### Correlation between modulation effect and lexical decision accuracy

**Fig. 3:**
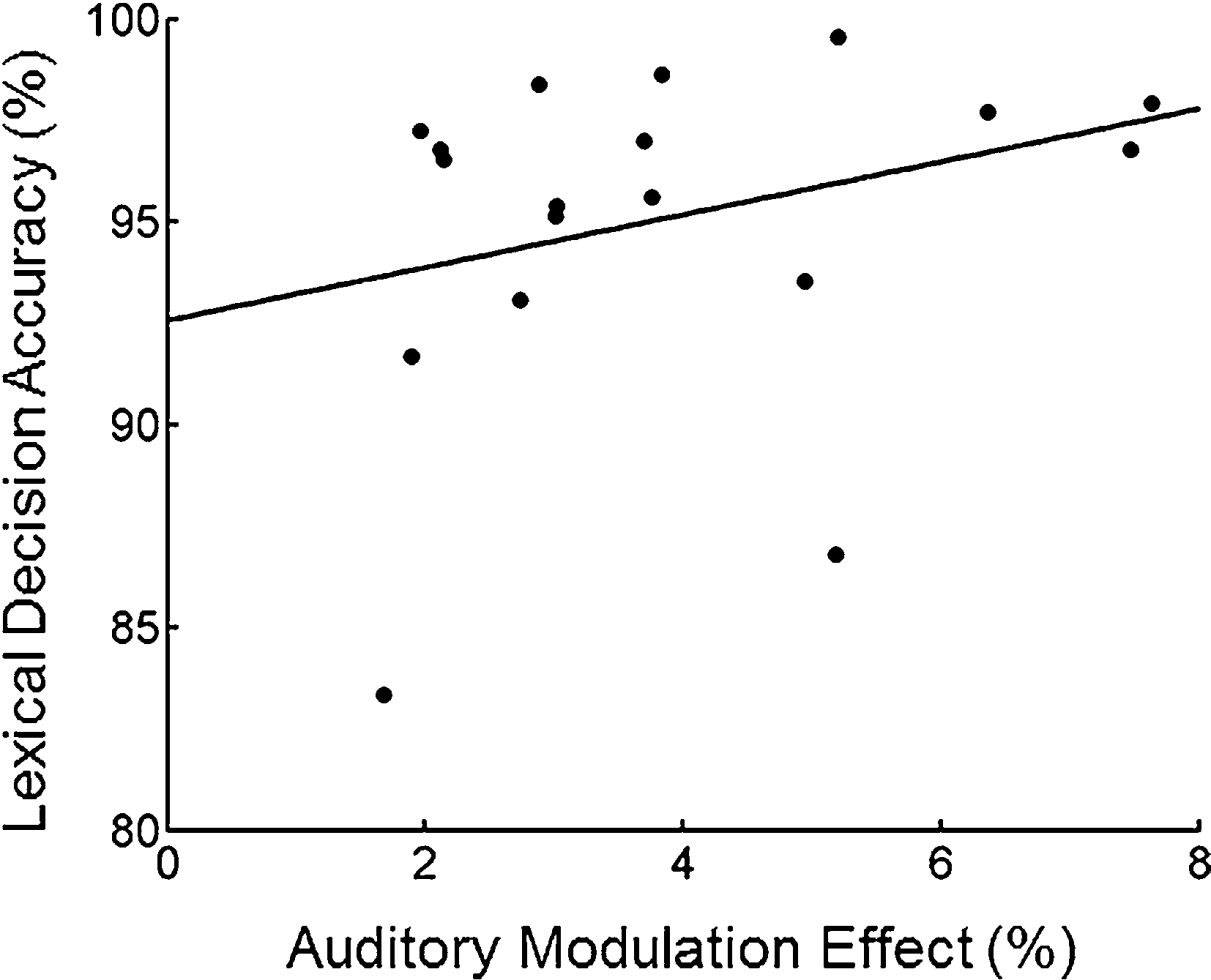
Significant correlation between auditory modulation effect and lexical decision accuracy (*r* = 0.47, *p* < 0.05) indicates higher modulation effect values coinciding with higher lexical decision performance. Robust correlation analysis (skipped Spearman correlation) showed that the correlation is still significant when the two outlier data points (the two *dots* below the data points cluster) are excluded from the analysis. For detail and the calculation of the modulation effect, please see the Methods section. The *solid line* shows a linear fit to the data

For each participant, we calculated a sound detection performance modulation effect indexing to what extent the detection performance benefitted from sound verb reading (see “Methods” section). A positive value is associated with an enhancement in sound detection performance after reading sound verbs and vice versa. Modulation effects ranged from −0.10 to 0.08, and 19 out of 29 participants had positive modulation effect values. From the high lexical decision performance group, 11 participants showed positive modulation effects. We further reasoned that within these 19 participants, better lexical decision accuracy should be associated with larger modulation effects because better lexical decision accuracy may indicate deeper word processing. This is indeed the case, as the modulation effect was significantly correlated with lexical decision accuracy (Spearman correlation coefficient = 0.47, *p* < 0.05, Fig. 3). Two bivariate outlier data points, i.e. the two participants with a lexical decision accuracy of less than 90 % shown in Fig. 3, were identified with the robust correlation analysis toolbox (Pernet et al. 2012). Leaving these two participants out, the correlation was still significant (skipped Spearman correlation coefficient = 0.47, bootstrapped 95 % confidence interval = [0.01 0.78]).

## Conclusions and discussion

We investigated the modulation of auditory perception during language comprehension using a sound detection task. The data showed that there in fact was modulation of auditory perception, but the effect depended on the participants’ performance in a lexical decision task. In the high-performance group, the group consisting of participants with a high accuracy in the lexical decision task, a clear modulation emerged. Sound detection accuracy was significantly higher after reading sound verbs compared to reading sound plus action verbs and abstract verbs. This modulation effect was not observed in the low-performance lexical decision group. RT as dependent measure did only show an effect of latency with shorter RTs in the long latency condition. A control analysis with lexical performance across word categories showed an interesting modulation effect in the LP group but not in the HP group.

Word frequency differences between word categories have principally to be taken into account, because they could contribute to the result. However, here word frequency is rather unlikely to contribute to the auditory modulation effect, as the only difference occurred was that sound plus action verbs have lower frequency than abstract words. Critically, there are no differences between sound verbs and sound plus action verbs or between sound verbs and abstract verbs, where auditory modulation effect was found. Furthermore, bigram frequency, trigram frequency, word length and subjective familiarity were controlled between word categories, which allow for an equal difficulty in the lexical decision task. These variables did not differ between conditions.

For both the short and the long latency conditions, we found a similar facilitation effect on sound detection performance from sound verb reading in the HP group. It should be noted that this does not contradict with the findings from motor studies, where an interference effect was found for short SOAs (100 ms) and a facilitation effect was found for long SOAs (350 ms) (de Vega et al. 2013). The short (50 ms) and long latencies (300 ms) in our study correspond to an SOA of 350 and 600 ms between the word onset and the response cue onset, respectively. Thus, both the short and long latencies should be viewed as long SOAs in the context of the study by de Vega et al. (2013). Very short SOAs (e.g. 100 ms) are not appropriate for the current study as in such case the stimulus presentation time is not long enough for participants to make meaningful lexical judgments. In the study by de Vega et al. (2013), the stimulus word was presented in the context of a sentence in which case prior predictions were available. Most importantly, the stimulus word continued to be present after the response cue onset. Future studies may combine this knowledge to investigate the effect of sound verbs on auditory performance in a shorter time scale.

While no significant interaction was found between latency (of tone presentation onset) and word category, the modulation effect was numerically stronger in the short latency condition, when the tone was presented 50 ms after the offset of the word (Fig. 2a). Interestingly, even in the LP group exhibiting no significant modulation effect, the pattern of accuracy values in the short latency condition resembled the pattern of the HP group (Fig. 2b). This may suggest that the modulation effect occurs with or just after reading words and decays gradually. This supports the idea that the modulation effect reflects language comprehension but not consecutive mental imagery (Kiefer et al. 2008; Klepp et al. 2014; Pulvermüller 1999; Trumpp et al. 2013b).

Word processing depth may be critical for the tone detection modulation effect. First, the modulation effect was only significant in the HP group but not in the LP group. The HP group participants may have been more attentive to the lexical decision task and thus processed the stimulus word in a deeper level. Second, the modulation effect was correlated with lexical decision accuracy across participants who showed a positive modulation effect. This may indicate that the deeper the word processing (as indexed by lexical decision accuracy), the stronger the modulation effect by word processing. We are aware that the above interpretation is speculative given the fact that we did not manipulate the word processing depth in the task. We propose that a semantic instead of a lexical decision task as we used here could produce an even stronger modulation effect than the one observed here. This would parallel the findings from language–motor interference studies (Sato et al. 2008).

In the case of abstract verbs, we interpret our findings as that the activation of auditory brain areas by sound verbs enhanced cortical excitability, thus leading to better perceptual performance compared with non-sound abstract verbs. In line with our prediction, sound detection accuracy after reading sound plus action verbs, which were both action and sound related, was lower than accuracy after reading sound verbs, which were sound related only. According to our hypothesis of combined effects of sound and action relatedness, we interpret this pattern of result as follows: sound relatedness of verbs, present in both sound and sound plus action verbs, enhances auditory performance. In the case of sound plus action verbs, the additional effect of action relatedness possibly inhibits auditory excitability and decreases performance in comparison to the sound verbs—in this case to a level comparable with the abstract verbs. Motor-induced suppression of auditory perception is a ubiquitous phenomenon across species (Crapse and Sommer 2008). In humans, it can even be observed during silent lip-reading (Kauramaki et al. 2010) or imagining speaking (Tian and Poeppel 2014). The finding here suggests that the comprehension of action-related verbs involves a simulation process including the activation of both motor and (auditory) sensory brain areas. One limitation of the current study is that we did not study the exclusive effect of action relatedness. This was because we were not able to collect a sufficient number of well matched purely action-related mouth verbs that are not associated with sound. Studies using other action- and sound-related verbs with the capability to disentangle motor and sound effects completely are needed to substantiate this finding.

Interestingly, our control analysis showed that in the LP group, participants were specifically impaired in processing sound verbs (lower lexical decision accuracy with sound verbs than with the other two verb categories). This pattern was not observed in the HP group. While the original idea of the study was to test the influence of word processing on auditory perception, the real scenario could be a bidirectional influence, i.e. word processing and auditory perception can influence each other (we thank anonymous reviewers for igniting the suggestion). The directionality of the influence may be related to participants’ strategy used in the task. When participants put more weight on the lexical decision task, the involvement of auditory cortex may have a positive effect in auditory perception (HP group). This also corresponds to our word processing depth hypothesis put forward earlier. In contrast, when participants put more weight on the auditory detection task, the involvement of the auditory cortex may have a negative effect in processing sound-related verbs (LP group) (Trumpp, Kliese et al. 2013). The RT data lend partial support for the idea that LP group participants put more weight on the sound detection task and that HP group participants put more weight on the lexical decision task. In the sound detection task, the LP group (mean = 628 ms; SD = 202 ms; averaged across all conditions) had numerically lower RTs than the HP group (mean = 663 ms; SD = 94 ms; averaged across all conditions) (see Fig. 2c, d). The RT in the lexical decision task was not recorded but can be estimated since the whole trial duration was recorded. The estimated RT data showed the opposite pattern to the RT data in the sound detection task, i.e. the HP group (mean = 722 ms; SD = 242 ms; averaged across all conditions) had numerically lower RTs than the LP group (mean = 855 ms; SD = 180 ms; averaged across all conditions). Unfortunately, a 2 (group) by 2 (task) mixed-design ANOVA analysis with the RT data only led to a significant main effect of task (task: *F*(1,27) = 7.36, *p* = 0.01; group: *F*(1,27) = 1.14, *p* = 0.30; interaction: *F*(1,27) = 2.53, *p* = 0.12). Albeit interesting, the RT in the lexical decision task was not timely recognized as a potential interest when planning the study—participants were explicitly told that they can take their time for the lexical decision task. Nevertheless, the reversal pattern of RT was reported having future studies in mind.

In conclusion, our study supports the view of embodied language comprehension, focussing on the auditory system and auditory contents of verbs. We have shown convergent evidence of auditory perception modulation after word reading in terms of enhancing behavioural performance. In addition to the finding that sound relatedness in words facilitates auditory perception, the study also suggests an interference effect on auditory perception from action relatedness in words. This extends our understanding of embodiment in language processing, taking into account different modalities. Unexpected (interesting) results from the lexical decision accuracy data further complement the embodied language comprehension view, suggesting a negative effect on sound verb processing from the auditory task.

## Appendix

Word stimuli (German) used in this study. English translations are included in the parentheses.

**Table:**

Real words			Pseudowords		
Sound verb	Sound + action verb	Abstract verb	Sound verb	Sound + action verb	Abstract verb
Brodeln (to seethe)	Husten (to cough)	Bessern (to improve)	Sposeln	Huseln	Bekkern
Hallen (to resound)	Keuchen (to pant)	Folgern (to conclude)	Rellen	Kenchen	Lelgern
Prasseln (to patter)	Kreischen (to screech)	Irren (to err)	Knosseln	Flieschen	Ürben
Rauschen (to whoosh)	Quengeln (to whine)	Mogeln (to cheat)	Fieschen	Drengeln	Soseln
Ticken (to tick)	Röcheln (to wheeze)	Schummeln (to cheat)	Nokken	Lücheln	Schunneln
Tosen (to roar)	Schluchzen (to sob)	Sehnen (to yearn)	Soden	Schmucknen	Särnen
Tuckern (to chug)	Schwatzen (to gabble)	Täuschen (to fool)	Huhsern	Schletzen	Telschen
Zirpen (to chrip)	Stöhnen (to groan)	Trotzen (to defy)	Zaspen	Stölzen	Kretzen
Zwitschern (to twitter)	Stottern (to stutter)	Wundern (to marvel)	Zwarschern	Stürtern	Fursern
Donnern (to thunder)	Schreien (to cry)	Büffeln (to swot)	Tennern	Schwieen	Güpfeln
Klingen (to chink)	Grölen (to bawl)	Grübeln (to brood)	Spinken	Frülen	Fröbeln
Klirren (to clank)	Johlen (to yell)	Hadern (to quarrel)	Spörren	Järlen	Wasern
Krachen (to crack)	Mampfen (to chomp)	Hassen (to hate)	Flasten	Lempfen	Wossen
Plätschern (to dabble)	Prusten (to snort)	Schulden (to owe)	Blotschern	Ruschen	Schunsen
Schallen (to echo)	Schnalzen (to chirrup)	Zaudern (to tarry)	Schakken	Schralben	Ziesern
Surren (to buzz)	Schnaufen (to wheeze)	Zweifeln (to doubt)	Huhben	Schraumen	Preimeln
